# Nutrition and Chronic Conditions

**DOI:** 10.3390/nu11020459

**Published:** 2019-02-22

**Authors:** Omorogieva Ojo

**Affiliations:** Faculty of Education and Health, University of Greenwich, London SE9 2UG, UK; o.ojo@greenwich.ac.uk; Tel.: +44-020-8331-8626; Fax: +44-020-8331-8060

**Keywords:** nutrition, chronic conditions, type 2 diabetes, cardiovascular diseases, cognitive decline, metabolic syndrome, pre-diabetes

## Abstract

This editorial discusses and analyses the role of dietary interventions in the management of chronic conditions in recognition of the global increase of these diseases, the rise in the ageing population, and the significant cost to health services around the world. Evidence has shown that low-glycaemic index (GI) and low-carbohydrate diets are effective in the management of type 2 diabetes, and the role of unsaturated fatty acids, vitamins, and bioactive compounds in chronic disease management have been the subject of intense research. However, although multidimensional approaches are important in the management of these chronic conditions, nutritional interventions are critical and central to these strategies.

Chronic conditions are diseases of long-term duration and may result from a combination of genetic, physiological, environmental, and behavioural factors [1]. The main types of chronic disease include cardiovascular diseases (which account for 17.9 million deaths globally every year), cancers (which are responsible for 9 million deaths annually), chronic respiratory diseases (3.9 million deaths/year), and diabetes (1.6 million deaths/year) [1]. In addition, mortality resulting from dementia more than doubled between 2000 and 2016, and it was the fifth leading cause of death worldwide in 2016 [2]. The increasing prevalence of these diseases is having a huge financial impact on healthcare systems globally and is arousing the attention and interest of researchers and policymakers at all levels of governance. With respect to diabetes, there were about 422 million adults who were living with the condition in 2014 [3]. This is significantly higher than the 108 million in 1980, representing a worldwide increase in diabetes prevalence from 4.7% in 1980 to 8.5% in 2014 among the adult population [3].

Strategies for managing these chronic conditions are usually multidimensional, and at the centre of these approaches are nutritional and/or dietary interventions, regular physical activity, and lifestyle modifications [3,4]. The role of nutrition in chronic disease management is particularly crucial as diet is a modifiable risk factor for most chronic conditions that exist either as single conditions or in comorbid states.

In this regard, Ojo et al. [5] conducted a systematic review and meta-analysis of randomised controlled trials with the aim of evaluating the effect of dietary glycaemic index (GI) on glycaemia in patients with type 2 diabetes. Six studies that met the inclusion criteria were selected for the meta-analysis. The results showed that, for the meta-analysis and sensitivity tests, there were significant differences (*p* < 0.001 and *p* < 0.001, respectively) between the low-GI diet and the higher-GI diet with respect to glycated haemoglobin in patients with type 2 diabetes [5]. Significant differences (*p* < 0.05) were also observed in relation to fasting blood glucose between the low-GI diet and the higher-GI diet. Therefore, it was concluded that a low-GI diet is more effective in managing blood glucose parameters (glycated haemoglobin and fasting blood glucose) than a higher-GI diet in patients with type 2 diabetes [5]. This conclusion is in line with the findings of an earlier systematic review by Thomas and Elliot [6] which showed that low-GI diets may promote glycaemic control in patients with diabetes. It is possible that because low-GI foods—including legumes, lentils, and oats—are made up of carbohydrates, which break down slowly in the gut and are absorbed slowly, may explain the findings of these reviews [6,7].

Apart from the role of a low-GI diet in the management of diabetes, the effect of a low-carbohydrate diet on glycaemic control in patients with type 2 diabetes has been explored [8]. The 49 patients who completed the study by Wang et al. [8] were randomly assigned to a low-carbohydrate diet encompassing an educational six-point formula (n = 24) and a low-fat diet with an educational six-point formula (n = 25). The findings of the study demonstrated that a low-carbohydrate diet can improve blood glucose more than a low-fat diet in Chinese patients with type 2 diabetes [8]. This result may be due to the decreased level of high-GI diets and the increased intake of nuts, which could improve insulin sensitivity and hyperglycaemia in the patients on the low-carbohydrate diet [9,10,11].

In another study, Perez-Leon et al. [12] examined the perspectives on the value of food and the challenges of dietary change among participants with type 2 diabetes and hypertension in four rural communities in Northern Peru. The study revealed the significant influence of culture and social conditions on food perceptions and dietary changes in rural Peru. It also showed that poor people in rural Peru make decisions and have knowledge relating to food that are based on experience, learning, and availability, which further underscores the multidimensional approach to chronic disease prevention and management [12].

Also of importance is the role of diet and inflammation in the chronic disease process. Inflammation, which is often characterised by pro-inflammatory markers such as interleukin-6 and tumour necrosis factor alpha, has been associated with type 2 diabetes [13,14]. Therefore, Denova-Gutierrez et al. [13] assessed the relationship between the dietary inflammatory index and the prevalence of type 2 diabetes among the adult population in Mexico City. A total of 1174 participants were involved in the study, and the data from a semi-quantitative food questionnaire were used to calculate the dietary inflammatory index scores for each of the subjects [13]. In addition, the criteria for establishing the participants with diabetes were clearly defined. The result of the survey showed that a pro-inflammatory diet was associated with significantly higher chances of type 2 diabetes in Mexican adults, which often manifests as chronic hyperglycaemia [13].

There is evidence that chronic hyperglycaemia in patients with diabetes can lead to short- and long-term complications, which may have serious implications for patients with diabetes. The long-term complications could be in the form of kidney dysfunction and cardiovascular diseases. In this regard, Sanjeevi et al. [15] examined the relationship between cardiovascular disease parameters and overall diet quality based on the Healthy Eating Index-2015. Altogether, 136 participants with type 1 diabetes were enrolled in this 18-month-long study, which was a secondary analysis of a randomised controlled trial of a behavioural nutrition intervention. The study revealed that the consumption of specific dietary components such as whole grains and polyunsaturated fatty acids may have an effect on cardiometabolic parameters in patients with type 1 diabetes, independent of blood glucose control [15].

In patients with prediabetes, early dietary interventions are critical in reducing the risk of developing type 2 diabetes. Wilson et al. [16] investigated the effect of consuming two SunGold kiwifruit per day on vitamin C levels, anthropometric and clinical parameters, and faecal microbiota in participants with prediabetes over a 12-week period. This was in recognition of the fact that kiwifruit is nutrient dense and is a major source of vitamin C. There is also evidence that supplementation of the diet with kiwifruit not only promotes plasma vitamin C but also reduces insulin resistance and enhances glycaemic control [16,17,18]. The results of the study showed a significant reduction in diastolic (4 mmHg; *p* = 0.029) and systolic (6 mmHg; *p* = 0.003) blood pressure and waist circumference (3.1 cm; *p* = 0.001) between baseline and the end of the study [16]. In addition, there was a significant decrease in glycated haemoglobin (1 mmol/mol; *p* = 0.005) and a significant decrease in fasting glucose (0.1 mmol/L; *p* = 0.046), although these differences were not clinically significant [16].

In a separate study, Alfawaz et al. [19] conducted a randomised controlled study to evaluate the differences between the effects of general advice on lifestyle changes, intensive lifestyle modification programmes, and general advice plus metformin in reducing the prevalence of metabolic syndrome in participants with prediabetes. The results demonstrated that full metabolic syndrome in the intensive lifestyle modification programme group decreased by 26% (*p* < 0.001) compared with 22.4% (*p* = 0.01) in the general advice and metformin group and 8.2% (*p* = 0.28) in the general advice alone group [19]. This study concluded that an intensive lifestyle modification programme was useful in reducing metabolic syndrome in Saudi subjects with elevated fasting glucose compared with other diabetes prevention programmes [19].

Sanchez-Rodriguez et al. [20] also examined the effects of virgin olive oils based on the contents of their bioactive compound on metabolic syndrome and endothelial function in healthy adults. Evidence from previous studies and reviews have shown that olive oil is a good source of monounsaturated fatty acids and bioactive compounds, including phenols [20,21,22]. In addition, it has been demonstrated that a Mediterranean diet supplemented with virgin olive oil may exert beneficial effects in people with cardiovascular disease in populations who are at high risk of developing the condition [20,21,22]. In the study by Sanchez-Rodriguez et al. [20], it was shown that olive oil rich in polyphenols increased high-density lipoprotein levels in females, although no differences were observed at the end of the interventions.

In addition to the role of monounsaturated fatty acids and polyphenols in chronic diseases, the effect of vitamin D in type 2 diabetes continues to gain the attention of researchers. This is because there appears to be evidence of an inverse association between levels of vitamin D (25-hydroxycholecalciferol) and the risk of developing type 2 diabetes among patients who are in a prediabetes state [23]. Therefore, Szternel et al. [24] explored the association between vitamin D status and the prevalence of dyslipidemia and impaired fasting glucose in children. It was concluded that vitamin D deficiency in children aged 9–11 years may have a negative effect on fasting glucose and total cholesterol concentration, and that children who are deficient in vitamin D are twice as likely to develop prediabetes than those with vitamin D greater than 20 ng/mL (50 nmol/L) [25].

Bruins et al. [25] also reviewed the role of nutrients in reducing the risk of noncommunicable diseases during ageing given the global increase in life expectancy. In addition, the authors noted that these chronic conditions such as diabetes, musculoskeletal disorders, cardiovascular diseases, and neurological disorders often increase with age. Bruins et al. [25] evaluated a range of evidence relating to the roles of vitamins D and K in musculoskeletal health, and the association between B vitamins, vitamins C, D, and E, and also omega-3 polyunsaturated fatty acids in older adults and their cognition. The effects of the micronutrients and macronutrients on cardiovascular diseases were also assessed [25]. Based on this review, Bruins et al. [25] revealed that inadequate intake of nutrients is common in older adults and that these represent a risk for the development of chronic diseases during ageing.

In another review, Klimova and Valis [26] sought to explore different types of nutritional interventions and their impact in preventing and delaying cognitive decline in healthy older adults. This is based on the increasing number of older people around the world, and the association between the ageing process and cognitive decline which manifests in the form of memory loss and attention deficit [26]. Twelve articles that met the inclusion criteria were included in the review. The nutritional interventions involved dietary supplements rich in fish oil or omega-3 fatty acid, vegetables such as avocado, berry and orange beverages, and Mediterranean diets. The findings of the review demonstrated that nutritional interventions have positive effects on cognitive function in healthy older adults [26]. In particular, it was suggested that interactions between more than one nutrient, as occurs in the Mediterranean diet, appear more effective than individual nutrients.

Although it is important to evaluate the role of nutrition in metabolic diseases such as diabetes and other long-term conditions that impact on memory and cognition, acne is a common chronic inflammatory skin disease that mostly affects adolescents, and the effect of nutrition in the pathophysiology of this condition is an interesting area of research [27]. For example, it is unclear whether there is a causal association between milk intake and acne. Therefore, Juhl et al. [28] explored the long-term effect of milk intake on acne in 20,416 Danish adults. This was a cross-sectional population study that involved the use of questionnaires to investigate milk intake. Juhl et al. [28] concluded that in the Danish general suburban population of adults, there was no observational or genetic association between milk intake and acne. In another article, Juhl et al. [29] conducted a systematic review and meta-analysis of 78,529 children, adolescents, and adults in an attempt to investigate the relationship between dairy intake and acne vulgaris. Fourteen articles were included in this review. Juhl et al. [29] showed that all dairy, including milk, yoghurt, and cheese, was found to be associated with an increased odds ratio for acne in participants who were 7–30 years of age. These findings appear to be in contrast with the results of the previous study [28], although in the latter review, Juhl et al. [29] noted that the results of the review should be interpreted with caution due to the level of heterogeneity and bias in the studies selected.

Knowledge of food consumption is also relevant when assessing the effectiveness of nutritional interventions in chronic conditions. Leyvraz et al. [30] noted that a high intake of salt was a major risk factor in the development of hypertension and cardiovascular diseases, and observed that improving the knowledge, attitudes, and practices in relation to salt intake was a useful strategy in mitigating the impact of these chronic diseases. Thus, Leyvraz et al. [30] conducted a survey involving 588 participants aged 25 to 65 years in five sub-Saharan African countries, namely Benin, Guinea, Kenya, Mozambique, and Seychelles. The essence of the study was to describe and compare the knowledge, attitudes, and practices of adults in relation to salt intake in these countries. The results showed that 85% of the participants were aware that a high salt intake could cause health problems, and 91% recognised the importance of limiting salt intake. In addition to these findings, Leyvraz et al. [30] recommended the need for education campaigns to reduce the intake of salt, including the salt content of manufactured food.

While dietary interventions are important in managing diabetes and other chronic conditions, the validity of the questionnaires used as tools for evaluating these interventions is very crucial. Collese et al. [31] conducted a systematic review and meta-analysis to establish the validity of questionnaires assessing fruit and vegetable consumption in children compared with blood biomarkers. Only two studies met the inclusion criteria and were included in this review, which may affect the wider application of the findings. Collese et al. [31] found that the use of questionnaires for assessing fruit and vegetable intake in children has fair criterion validity.

In another review, Sugizaki and Naves [32] evaluated the potential prebiotic properties of nuts and edible seeds, and their relationship to obesity. Obesity is a complex and significant public health challenge, including being a risk factor for type 2 diabetes and cardiovascular diseases. Based on this review, Sugizaki and Naves [32] proposed three mechanisms to explain the potential role of nuts and edible seed consumption in intestinal homeostasis and body weight control. These include the maintenance of enteric barrier integrity, improvement of anti-inflammatory status, and enhancement of butyrate synthesis.

Based on the above, it is clear that multidimensional approaches are essential in the management of chronic conditions, and nutritional interventions are critical and central to these strategies. There is evidence that demonstrates the effectiveness of low-GI and low-carbohydrate diets in the management of type 2 diabetes, and the role of unsaturated fatty acids, vitamins, and bioactive compounds in chronic disease management.
